# Role of Incretin Axis in Inflammatory Bowel Disease

**DOI:** 10.3389/fimmu.2017.01734

**Published:** 2017-12-06

**Authors:** Lihua Duan, Xiaoquan Rao, Zachary Braunstein, Amelia C. Toomey, Jixin Zhong

**Affiliations:** ^1^Department of Rheumatology and Clinical Immunology, The First Affiliated Hospital of Xiamen University, Xiamen, China; ^2^Cardiovascular Research Institute, Case Western Reserve University, Cleveland, OH, United States; ^3^Boonshoft School of Medicine, Wright State University, Dayton, OH, United States; ^4^Department of Health Sciences, University of Missouri, Columbia, MO, United States

**Keywords:** incretin, inflammatory bowel diseases, dipeptidyl peptidase-4, glucagon-like peptide-1, liraglutide

## Abstract

The inflammatory bowel diseases (IBDs), including Crohn’s disease (CD) and ulcerative colitis (UC), are chronic inflammatory conditions of the gastrointestinal tract and involve a complicated reciprocity of environmental, genetic, and immunologic factors. Despite substantial advances in the foundational understanding of the immunological pathogenesis of IBD, the detailed mechanism of the pathological progression in IBD remains unknown. In addition to Th1/Th2 cells, whose role in IBD has been previously well defined, recent evidence indicates that Th17 cells and Tregs also play a crucial role in the development of IBD. Diets which contain excess sugars, salt, and fat may also be important actors in the pathogenesis of IBD, which may be the cause of high IBD incidence in western developed and industrialized countries. Up until now, the reason for the variance in prevalence of IBD between developed and developing countries has been unknown. This is partly due to the increasing popularity of western diets in developing countries, which makes the data harder to interpret. The enterocrinins glucagon-like peptides (GLPs), including GLP-1 and GLP-2, exhibit notable benefits on lipid metabolism, atherosclerosis formation, plasma glucose levels, and maintenance of gastric mucosa integrity. In addition to the regulation of nutrient metabolism, the emerging role of GLPs and their degrading enzyme dipeptidyl peptidase-4 (DPP-4) in gastrointestinal diseases has gained increasing attention. Therefore, here we review the function of the DPP-4/GLP axis in IBD.

## Introduction

Inflammatory bowel diseases (IBDs), including Crohn’s disease (CD) and ulcerative colitis (UC), are chronic intestinal inflammatory conditions that might be caused by environmental, genetic, and immunological imbalances (1–3). The clinical treatments for these diseases are very limited and inefficient (4, 5). To develop novel therapeutic strategies for IBD, enormous research has been focused on exploring the detailed mechanism of IBD pathophysiology. Animal models, including trinitrobenzene sulfonic acid (TNBS)-induced experimental colitis, dextran sulfate salt (DSS)-induced colitis, and a number of genetic mouse models (such as IL-10^−/−^), have been established to study the underlying mechanisms (6).

It is well accepted that dysregulated immune response plays a critical role in colitis (7–10). Tumor necrosis factor-α (TNF-α) is a well-studied cytokine that is implicated in the pathological progression of human IBD. Inhibition of TNF-α activity by anti-TNF-α antibody has been widely used as a clinical treatment for IBD. Studies also indicate a profound role of the Th17/Treg axis in the pathogenesis of IBD (11, 12). Therefore, the immune suppressive drugs which can inhibit the effector T cells immune response and promoting Treg expansion are also being used in IBD patients. However, not all patients exhibit an effective response to this therapy (13, 14). In addition, serious side effects, including infection, anaphylaxis, and malignancy, have been observed during these treatments (15). Therefore, alternative therapeutics are imperative for the treatment of IBD.

Glucagon-like peptides (GLPs), including GLP-1 and GLP-2, are secreted by the endocrine cells in the gut up on nutrient uptake (16–18). Through stimulating the islet β cells to secret insulin, inhibiting gastric emptying, and reducing food ingestion, GLP-1 plays a crucial role in lowering blood glucose and controlling body weight (19, 20). Therefore, GLP-1 was used in human subjects with type 2 diabetes, especially in obese patients with type 2 diabetes (21, 22). In contrast, GLP-2 is used as a therapy for intestinal injury and short bowel syndrome due to its effects of promoting mucosal epithelium expansion, and crypt cell proliferation and improving intestinal adaptation and nutrient absorption (23–27). Because GLPs are degraded by dipeptidyl peptidase-4 (DPP-4) very quickly, resulting in very short half-lives (minutes) *in vivo* (28–31), the DPP-4 inhibitors have recently gained increasing attention (19, 21).

The role of incretin hormones in bowel disease has not been demonstrated until recently (32, 33). In DSS-induced colitis, the severity of intestinal injury was increased in GLP-1R^−/−^ mice (34). In consistency with this, administration of GLP-2 led to significant improvements in animal weight loss and intestinal inflammation in IL-10-deficient mice, a spontaneous colitis mouse model (35). Here, we will discuss in-depth the actions of DPP-4/GLP axis in IBD.

## Overview of GLP Function

Glucagon-like peptide-1 exerts pleiotropic function through binding to the GLP-1 receptor and is involved in the development and progression of many diseases (17, 18). The GLP-1 receptor is widely expressed in many organs and tissues, including the endocrine pancreas, gastrointestinal tract, heart, and central nervous system. More recent work has shown that a defect in cellular response to GLP-1, akin to insulin resistance, in combination with a diminishment of GLP-1, has a predominant role in the pathogenesis of patients with T2DM. Exogenous administration of pharmacological doses of GLP-1 receptor agonists have been shown to restore β-cell sensitivity to insulin and induce the secretion of insulin. Impaired incretin response is associated with insulin resistance in both non-diabetic and diabetic individuals (36, 37).

The first two amino-acid residues in the *N*-terminus of GLP-1 are His–Ala, which causes its susceptibility to DPP-4 degradation. The *N*-terminal His–Ala residues of GLP-1 are rapidly cleaved by DPP-4 expressed on surrounding tissues, resulting in the inactivation of GLP-1 (38). Exenatide, liraglutide, dulaglutide, albiglutide, and lixisenatide are structurally modified GLP-1 analogs used in the clinical setting, exhibiting relative resistance to the cleavage by DPP-4, and a long-circulating half-life (39). Exogenous GLP-1 administration potently inhibits gastric emptying in rodent and human studies, which favors body weight loss (40). Diabetic patients are prone to develop cardiac disorders; the actions of GLP-1 on cardiac function were investigated (41). Since GLP-1 receptor is widely expressed in the brain, the role of GLP-1 in central nervous system, beyond its regulatory function on glycemic control, was explored (42, 43). Expectedly, GLP-1 possesses a protective effect on neuronal damage by reducing ibotenic acid-induced depletion of choline acetyltransferase immunoreactivity (44). GLP-1 receptor-deficient mice were shown to have defects in cognitive function (45), synaptic plasticity, and memory formation (46), which are recovered by transferring the GLP-1R gene in the hippocampus (47). These data reveal that GLP-1 may have pleiotropic functions in a multitude of diseases. The actions of GLP-1 in IBD will be discussed below.

Glucagon-like peptide-2 is a 33 amino-acid peptide and that is cleaved by DPP-4 in rodents and humans, but with a half-life that is slightly longer than GLP-1 (17). Unlike GLP-1, which plays a role in glucose homeostasis, GLP-2 primarily exerts a potential effect in intestinal weight gain, mucosal development, and intestinal integrity (17, 27). In view of the above-mentioned effects, GLP-2 treatment reduced intestinal inflammation and improved intestinal healing after injury (48, 49). In addition to the benefits in improving intestinal integrity, GLP-2 also exhibits antimicrobial effects by regulating the synthesis and activity of Paneth cell-produced antimicrobial peptides (50). In addition, GLP-2 reduces bacterial invasion by promoting secretory immunoglobulin A (IgA) expression (51). Because GLP-2 receptor is widely expressed on many tissues and cells, physiological effects of GLP-2 beyond the gut have also been reported. Like GLP-1, GLP-2 also regulates the function of central nervous system (52). Activation of GLP-2 receptors can reduce stress-induced depression (53, 54) and improve memory in animal experiments (55). It also plays a substantial role in bone metabolism *via* reducing bone reabsorption and improving bone mineral density (56). GLP-2 has been shown to improve liver regeneration and enhance lung recovery in mice (57, 58). Thus, GLP-2 reveals potential effects in and out of the gastrointestinal tract.

## The Role of GLPs in Immune Regulation

Recent studies have demonstrated that GLPs exert inflammation regulatory functions in metabolic disease. Administration of GLP-1 markedly reduced the macrophage infiltration and the production of inflammatory cytokines in the adipose tissue in ob/ob mice (59). GLP-1 has also been shown to regulate invariant natural killer T cells (iNKT) and macrophage function in humans (60, 61). Animal study carried out in Glp1r^−/−^ mice suggested that GLP-1 may play a role in maintaining peripheral Treg numbers and suppressing lymphocyte hyperproliferation (62). Although GLP-2 can also blunt inflammatory cytokine production *via* inhibition of NF-κB activity and ERK phosphorylation (63), enhanced macrophage accumulation was observed in the colon of colitic mice (35). A recent study reported that GLP-1 controls of gut immunity by regulating the intestinal intraepithelial lymphocyte function, leading to a protective role in the DSS-induced colitis (34). In consistency, GLP-2 treatment also reduced pro-inflammatory cytokine protein levels in the IL-10-deficient mouse model of colitis (35). Taken together, GLPs play a crucial role in inflammation regulation and gut disorders.

## Physiological Role of DPP-4 Function

Dipeptidyl peptidase-4, a type-II integral transmembrane glycoprotein, is best known for its catalytic function. A soluble form of DPP-4, which lacks the cytoplasmic and transmembrane domain, with preserved catalytic activity is also detected in the plasma (38, 64). Although the mechanism of regulation of DPP-4 expression remains unclear, TNF-α has been implicated (65, 66). The primary substrates for DPP-4 are enterocrinins, such as GLP-1, GIP, and GLP-2, which are responsible for glucose metabolism (17, 39, 67). DPP-4 gene-deficient mice show improved postprandial glucose control and are resistant to the progression of obesity and hyperinsulinemia. Inhibition of DPP-4 enzymatic activity with pharmacological agent administration improves glucose tolerance in wild-type mice, but not in DPP-4 knockout mice (68).

In addition to enterocrinins, some chemokines and cytokines could also be cleaved by DPP-4, such as stromal cell-derived factor-1 (SDF-1, also known as CXCL12), G-CSF, IL-3, GM-CSF, and erythropoietin, thereby allowing DPP-4 to regulate immune responses (69). DPP-4 also exerts non-catalytic functions *via* interacting with adenosine deaminase (ADA), caveolin-1, fibronectin, and CXCR4 (70, 71). The best-known non-catalytic function is the interaction between DPP-4 and ADA, which can act as a co-stimulatory dyad to promote T-cell activation. Our previous work has demonstrated a role of DPP-4 non-enzymatic function in regulating dendritic cell (DC)/macrophage-mediated adipose tissue inflammation in obesity (64). We also showed that long-term DDP-4 inhibition reduces atherosclerosis and inflammation *via* effects on macrophage migration (CD11b^+^, CD11c^+^, and Ly6C^hi^) (72, 73). Furthermore, in non-obese diabetic (NOD) mice, DPP-4 inhibitors significantly increased the TGF-β levels and Treg expansion (74). Beyond that, our recent study, as well as others, demonstrated that DPP-4 plays a role in the infection of Middle Eastern respiratory syndrome (MERS) virus (75).

## Effects of GLP-1 on IBD

BP-lowering and anti-atherosclerotic effects of GLP-1R agonists have been well demonstrated, while the gastrointestinal effects of GLPs are underappreciated. Here, we will discuss the relationship between GLP-1 and inflammation in the gastrointestinal tract. UC patients with colectomy showed a slower release of GLP-1 in response to intake of glucose (76). Consistently, postprandial GLP-1 response was also impaired in patients with ileostomy (77). Yet it was not known whether the colectomy or inflammatory state affects the GLP-1 release in IBD. Subsequent studies demonstrated that although GLP-1r mRNA levels was reduced in samples harvested from inflamed sites of IBD patients and colitis mice (78), GLP-1 levels were increased in sera of IBD patients when compared with healthy controls (79, 80). The defective GLP-1 release in IBD patients with colectomy might be caused by the loss of the colonic endocrine tissue.

Therefore, these data reveal a link between gut inflammation and GLP-1 expression and brings up an emerging question that how GLP-1 is implicated in IBD. To explore this question, some studies were conducted in experimental animal colitis. In T-cell adoptive transfer-induced colitis, the GLP-1 expression in colonic tissue was significantly diminished in SCID mice with adoptive transfer of CD4^+^ T cell when compared with control mice (81). Furthermore, in DSS-induced colitis, a considerable increase of GLP-1 was detected in colitic mice with DPP-4 inhibitor treatment (82). Notwithstanding alteration of GLP-1 expression in colitis, the exact role of GLP-1in the development of colitis remains unknown, in terms of being beneficial or detrimental. A recent study showed that the GLP-1 analog liraglutide exerts a significant improvement of disease activity endpoints, including colonic tissues histological changes and colon weight/length ratio, which might be due to its role in reducing inflammatory cytokines and chemokines, such as chemokine (C–C motif) ligand 20 (CCL20), IL-33, and IL-22 (78). As has been previously established, CCL20 is a key chemokine for CCR6 + Th17 cells (83), while IL-33 and IL-22 are the representative cytokines for Th2 and Th17 immune responses, respectively (84, 85). In line with above results, GLP-1 in sterically stabilized phospholipid micelles (GLP-1-SSM), showing a long half-life and resistant to DPP-4, markedly alleviated the development of DSS-induced mice colitis by reducing the expression of pro-inflammatory cytokine IL-1β (86). Moreover, intestinal epithelial architecture in a colitis model with GLP-1-SSM administration was significantly improved. In conclusion, GLP-1 might act as a novel therapeutic tool in ameliorating colonic inflammation.

## The Inflammatory Regulation of GLP-2 on IBD

Regarding the inhibition of enterocyte apoptosis and stimulation of crypt cell proliferation, GLP-2 is thought to be associated with tissue repair during injury or infection (17, 23). Therefore, in chemically induced enteritis (48) or vascular-ischemia reperfusion injury (87–89), GLP-2 shows a protective effect based on reducing epithelial barrier damage and lowering bacterial infection. It stands to reason that GLP-2 might be a potential therapeutic target in IBD, a condition characterized by destruction of the gastrointestinal epithelium. In an adoptive CD4^+^ T-cell transfer model of colitis, the amount of GLP-2 in colon tissue was also further decreased compared with that in normal mice or SCID mice without CD4^+^ T-cell adoptive transfer (81). However, these results were not duplicated in human IBD samples. A study showed no changes of GLP-2 levels in fasting plasma between IBD patients and controls, which pinpoints L-cell secretion is not altered in the pathogenesis of IBD (90). Nevertheless, the circulating levels of bioactive GLP-2 (1–33) were markedly increased in CD and UC patients (91). The alteration of GLP-2 (1–33) might be due to an adoptive response to intestinal injury, which promotes mucosal epithelium restoration in a self-repair mechanism. The discrepant data might be the causal agent of the different inflammatory conditions, because an increase in GLP-2-immunoreactive L cells was found in remissive status of colitis. Another reason is probably due to the detection reagent which detects all GLP-2 or bioactive GLP-2 (1–33).

Beyond the promotion of crypt cell proliferation and mucosal integrity, GLP-2 also exerts a distinct role in anti-inflammatory actions. To mimic anti-inflammatory therapeutic approaches in humans, a combination of GLP-2 with aminosalicylates (ASAs) or corticosteroids were administrated into mice with DSS-induced colitis, while no synergistic effect was observed. Interestingly, corticosteroid administration prevented the intestinal weight increase when the mice were treated with corticosteroids and GLP-2 (92), while these treatments exhibited a similar anti-inflammatory effect in colonic tissues. However, in TNBS-induced ileitis and DSS-induced colitis, GLP-2 treatment downregulated expression of inflammatory cytokines, including IFN-γ, TNF-α, and IL-1β, while the anti-inflammatory cytokine IL-10 was increased (93). Another report also showed that GLP-2 alleviates the development of colitis through reducing the pro-inflammatory cytokines in IL-10-deficient mouse model. The level of inducible nitric oxide synthase (iNOS), a marker for classically activated macrophage, was reduced in GLP-2-treated mice (35). This suggests that GLP-2 might alter macrophage polarization.

It is noteworthy that chronic colitis is a risk factor for colon cancer. Interestingly, a few reports have shown that exogenous and endogenous GLP-2 is a potential cancer promoter in mice models, although reduced inflammation was also observed (94, 95). This might be resulted from the strong preference of GLP-2 for epithelium proliferation. Therefore, the surveillance of dysplasia and colon cancer must be vigilant in GLP-2 treatment.

## Inhibition of DPP-4 Function in IBD

Regarding a catalytic function of DPP-4 on GLP-1 and GLP-2, previous studies have demonstrated that DPP-4 can act as an immune regulator *via* its expression on immune cells and the ability to cleave biologically active chemokines and cytokines. Hence, DPP-4 involvement in the pathogenesis of colitis has been proposed (96). The involvement of DPP-4 might depend on two major pathways: the catalytic function and non-catalytic function (38, 73, 97). Like GLP-2, DPP-4 inhibitors have a proliferative effect on the colonic epithelium (98). It has also been demonstrated that the protective effects of DPP-4 inhibitors in IBD might be a result of increased levels of GLP-1 (82). Notably, plasma GLP-2 levels were increased in response to DPP-4 inhibitor. Thus, the effect on epithelium expansion induced by DPP-4 inhibitor probably relies on the indirect elevation of GLP-2 expression (99). To investigate the influence of DPP-4 in the pathogenesis of DSS-induced colitis, DPP-4-deficient mice were used in DSS treatment, and an increase of myeloperoxidase (MPO) activity and expression of NF-κB p65 subunit in the colonic tissues was observed. Furthermore, an increase in the percentage of splenic CD8^+^ cells and NKT cells in CD26-deficient mice was observed (100). In keeping with GLP-2-treated mice, DPP-4-deficient mice also showed a significant increase in macrophages when compared with wild-type mice (101). These data reveal a detrimental role of DPP-4 during the development of colitis. Conversely, DPP-4-deficient rats reveal an apparent diminished disease activity index (DAI) in the low-dose DSS-induced colitis, especially in 1% DSS-induced colitis (102). A similar effect was also investigated in DPP-4 inhibitor anagliptin- and ER-319711-treated mice with DSS-induced colitis (98). In addition to ER-319711, anagliptin administration ameliorated the body weight loss and DAI. Additionally, a significantly lower histological score was observed in the anagliptin-treated group (103), which suggests that inhibition of the DPP-4 activity can facilitate the resolution of mucosal damage. Taken together, these findings suggest a complex and dichotomous biology during the development of IBD, which might be due to its multifunction.

## Conclusion

**Figure 1:**
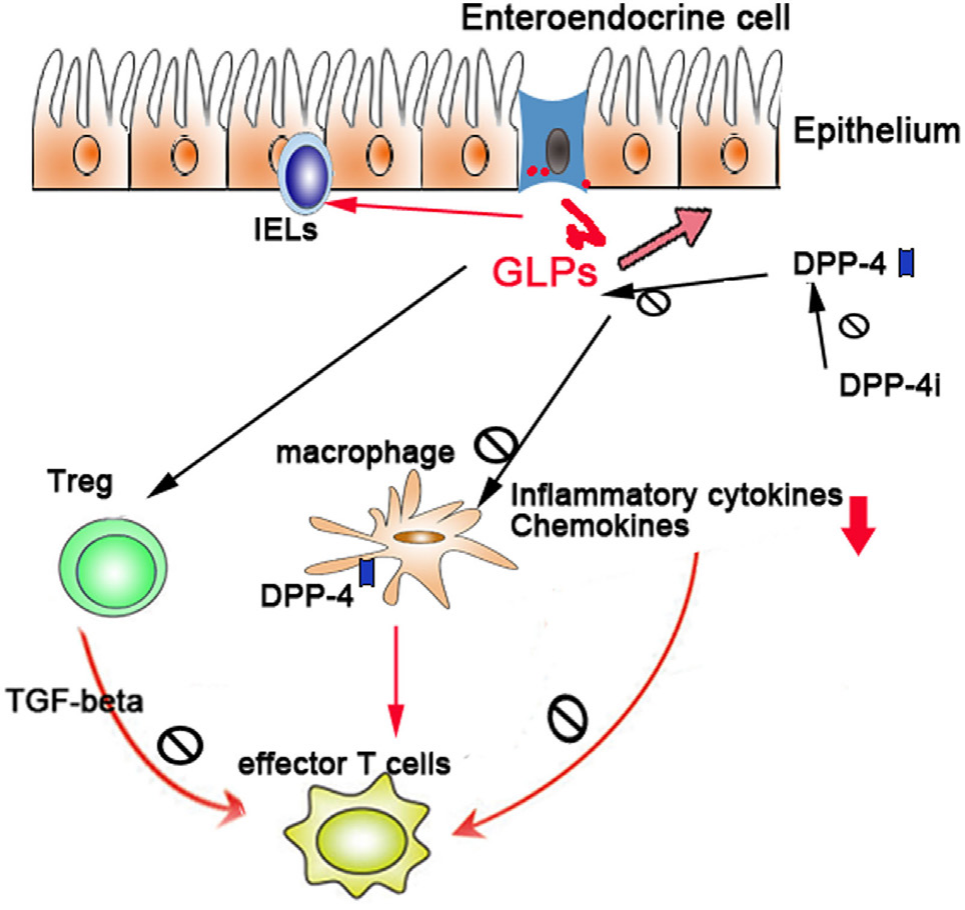
The regulatory role of dipeptidyl peptidase-4 (DPP-4)/glucagon-like peptides (GLPs) in gut immunity: DPP-4/GLP axis regulates gut inflammation through (1) promoting the tissue repair of injured epithelium, (2) regulating the differentiation and functions of Treg and intraepithelial lymphocytes, and (3) regulating the function of macrophages and dendritic cells.

Due to the vital role of GLPs in intestinal healing and anti-inflammatory function, a sound understanding of the production, regulation, and function of GLPs and their degrading enzyme DPP-4 will facilitate the treatment of colitis. The potential mechanisms (Figure 1) of DPP-4/GLP axis in the IBD may include the following: (1) GLPs promote the tissue repair of injured epithelium; (2) GLPs regulate T-cell differentiation and functions (e.g., Treg, effector T cells, and intraepithelial lymphocytes); (3) GLPs and DPP-4 regulate the function of innate immune cells such as macrophages and DCs; and (4) suppression of DPP-4 enzymatic activities by pharmacological inhibitors preserves GLP function. Although most studies in this area mainly were carried out on animal models and there are limited clinical trials, a phase-II clinical trial of teduglutide (a GLP-2 analog) observed a remission rate of 55.6% in CD patients (104). To what extent GLPs and DPP-4 contributes to IBD in humans requires further investigation.

## Author Contributions

LD and XR reviewed the literature and wrote the first draft. ZB, AT, and JZ reviewed the literature and finalized the manuscript. All authors have read and approved the final manuscript.

## Conflict of Interest Statement

JZ is currently receiving a grant from Boehringer Ingelheim (IIS2015-10485). The remaining authors have no conflicts of interest.
